# Two‐ and Three‐dimensional Transthoracic Echocardiographic Identification of Esophageal Stent

**DOI:** 10.1111/echo.70080

**Published:** 2025-01-20

**Authors:** John LaForge, Donovon Allen, Regina Dickey, Navin Nanda

**Affiliations:** ^1^ Department of Internal Medicine University of Alabama at Birmingham Birmingham Alabama USA; ^2^ Echocardiography Laboratory University of Alabama at Birmingham Birmingham Alabama USA; ^3^ Cardiology Division University of Alabama at Birmingham Birmingham Alabama USA

**Keywords:** esophageal stent, two‐dimensional transthoracic echocardiogram, three‐dimensional transthoracic echocardiogram

## Abstract

First echocardiographic detection of esophageal stent.

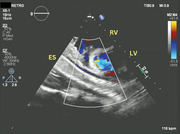

1

The patient was a 39‐year‐old male with extensive esophageal stenosis due to squamous cell carcinoma with metastatic disease to bone, lymph nodes, and pleura presenting with dysphagia and fever [1]. To assist with his dysphagia, a palliative 20 mm × 125 mm partially covered, Evolution (Cook Medical LLC, Bloomington, Indiana, USA) controlled‐release stent was placed spanning from mid esophagus to the proximal stomach [2]. Because of the above findings and to rule out endocarditis, two‐dimensional transthoracic echocardiography (2DTTE) using a Philips (Andover, Massachusetts, USA) EPIQ ultrasound equipment with a matrix 5‐1MHz transducer was done. 2DTTE demonstrated a posteriorly located stent that was identified as a long tubular structure with closely packed linear echogenic striations consistent with the nitinol meshwork present in the stent. Small echo densities moving within the stent were also visualized similar to particulate matter previously observed by us in the inferior vena cava [3]. The etiology is unknown but, in our patient, these may be related to tissue coming through the mesh of the stent. Swallowed saliva and cardiac motion may also have a role in this (Figure 1 and Video S1A–D). Three‐dimensional transthoracic echocardiography (3DTTE) was also performed, using the same transducer as 2DTTE, and showed findings similar to 2DTTE. Additionally, linear structures attached to the stent wall were noted by 3DTTE which could be due to tumor or other tissue infiltration. A longer length of the curved stent together with more extensive nitinol meshwork were also visualized with 3DTTE as compared to 2DTTE (Figure 2, Video S2A,B). These findings related to the ability of cropping the 3DTTE datasets in any desired manner resulting in a more comprehensive assessment of the esophageal stent compared to 2DTTE. Both 2DTTE and 3DTTE showed a small pericardial effusion but were otherwise normal with no evidence of left and right ventricular dysfunction and no vegetations. The esophagus is not normally identified by echocardiography since it is normally closed and even if open during swallowing the air within would preclude imaging by ultrasound. Computed tomography scans (CTs) done in our patient are shown in Figure 3A,B. This case draws echocardiographers’ attention and awareness to the ability of echocardiography to image as well as render identification of an esophageal stent on TTE. This can be done by noting the similarity of its location to the expected anatomic position of the esophagus with its relationship to surrounding structures such as the descending thoracic aorta and the vertebral column (Video S1C). Presence of particulate matter further facilitates its identification. Since, to the best of our knowledge, this is the first case demonstrating an esophageal stent by echocardiography, there are no studies showing its clinical usefulness. However, echocardiography is cheaper than CTs, radiation‐free, and in selected cases may have potential in assessing esophageal stent complications such as changes in stent position or shape, stent breakage, and tumor infiltration, especially in those with absolute or relative contraindications to CTs and follow‐up studies [2].

**FIGURE 1:**
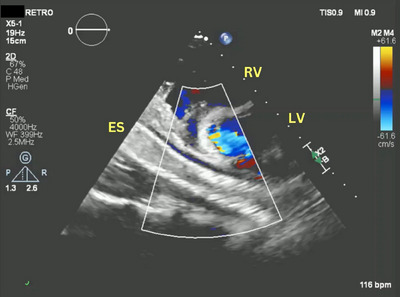
Two‐dimensional transthoracic echocardiography. Off axis short axis view. The ES is visualized in long axis posterior to LV and right RV. ES, esophageal stent; LV, left ventricle; RV, right ventricle.

**FIGURE 2:**
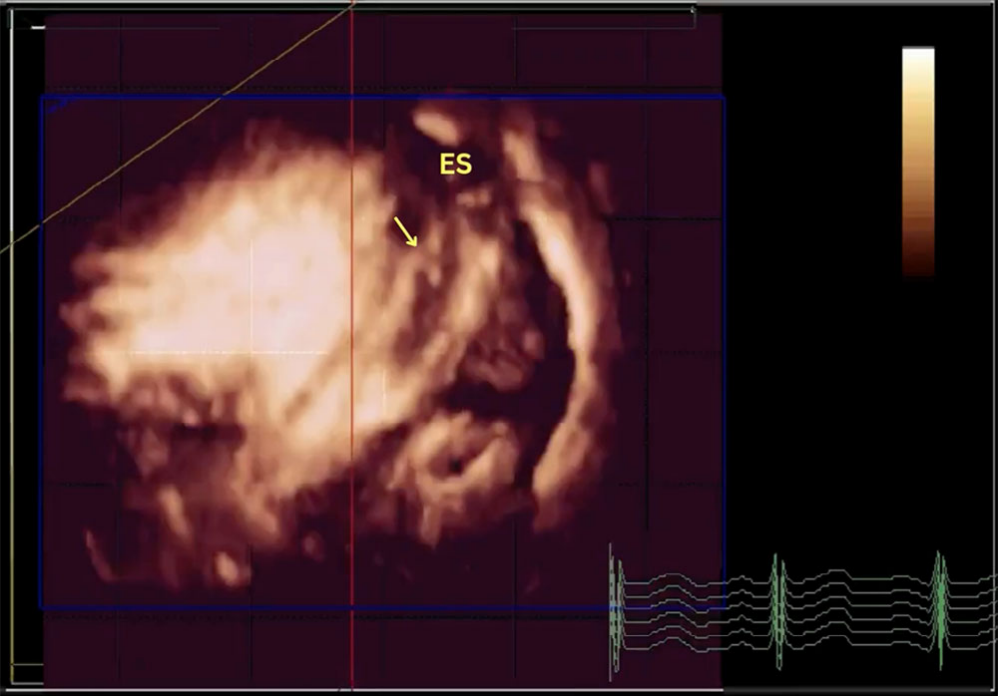
Three‐dimensional transthoracic echocardiography. Full volume mode. The arrow points to a linear structure hanging down from the ES wall. ES, esophageal stent.

**FIGURE 3:**
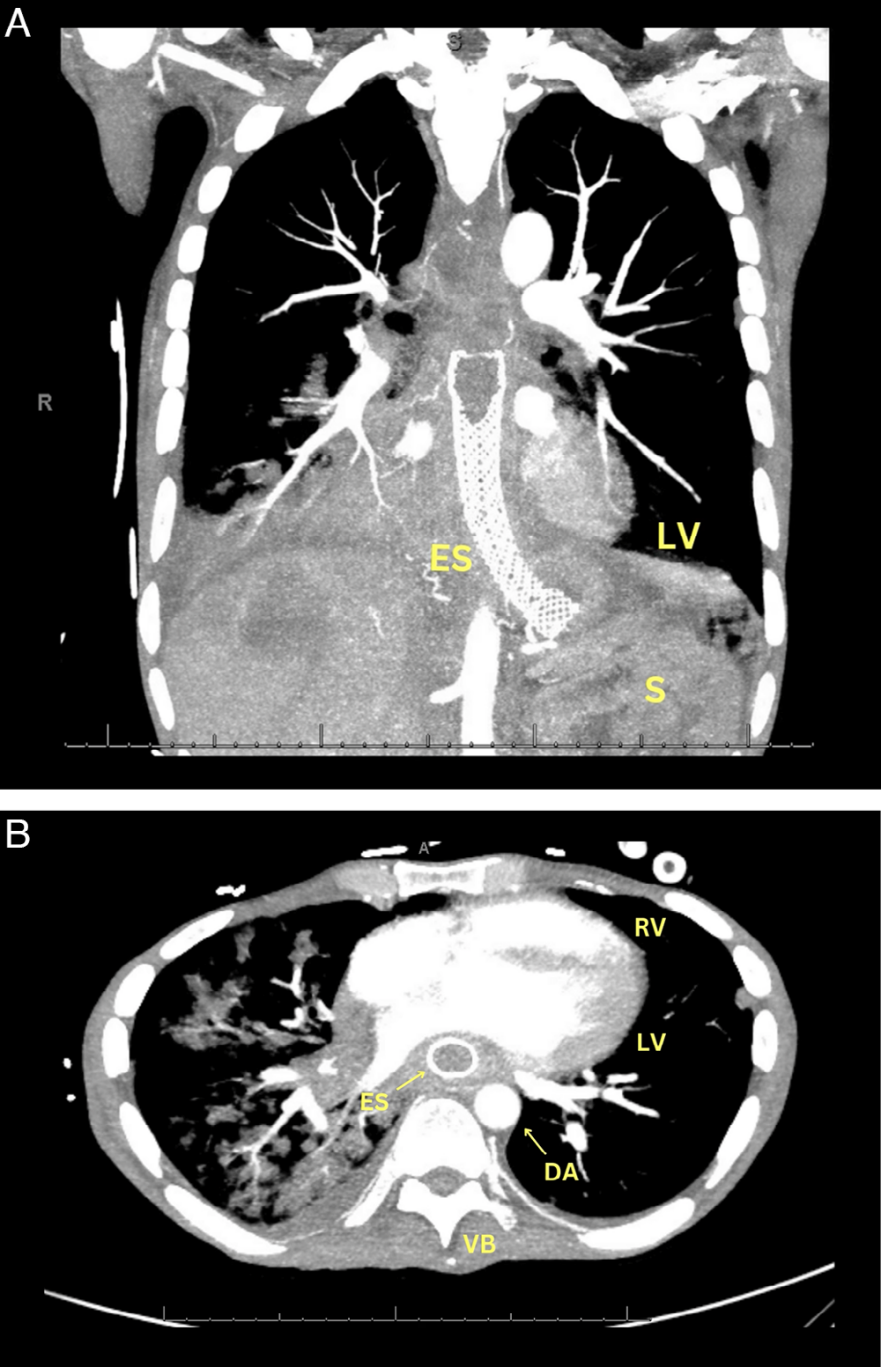
Computed tomography scans. (A) Shows the esophageal stent with the nitinol meshwork. (B) Shows the relationship of the ES to other anatomic structures. ES, esophageal stent; S, spleen. Other abbreviations are in Figure 1.

## Supporting information



Supporting Information

Supporting Information

Supporting Information

Supporting Information

Supporting Information

Supporting Information

Supporting Information

## References

[1] J. K. Waters and S. I. Reznik , “Update on Management of Squamous Cell Esophageal Cancer,” Current Oncology Reports 24, no. 3 (2022): 375–385, 10.1007/s11912-021-01153-4. 35142974

[2] P. Hindy , J. Hong , Y. Lam‐Tsai , and F. Gress , “A Comprehensive Review of Esophageal Stents,” Gastroenterology Hepatology 8, no. 8 (2012): 526–534. 23293566 PMC3533211

[3] E. K. Kerut , M. Dearstine , P. Dottery , and N. C. Nanda , “Particulate Matter Within the Inferior Vena Cava,” Echocardiography 25, no. 7 (2008): 803–804, 10.1111/j.1540-8175.2008.00733.x. 18754941

