# Supplementary material for: Two‐ and Three‐dimensional Transthoracic Echocardiographic Identification of Esophageal Stent
Source: Echocardiography. 2025 Jan 20;42(1):e70080. doi: 10.1111/echo.70080 (PMC11745407; doi:10.1111/echo.70080)
Supplement: Supplementary file 7 — Supporting Information [file ECHO-42-e70080-s003.docx]

Video 1A corresponds to Figure 1.

Video 1B. Subcostal view. Shows particulate matter in the stent. L=liver.

Video 1C. Parasternal long axis view. Shows the stent in short axis. AO=aorta; DA=descending thoracic aorta; VB= shadowing from vertebral body. The arrow points to the coronary sinus.

Video 1D. Subcostal view. Shows particulate matter in the stent. L=liver; VB= shadowing from vertebral body; PE=pericardial effusion.

Video 2A corresponds to Figure 2.

Video 2B shows extensive ES meshwork.
